# Q&A - Economic analyses for vaccine introduction decisions in low- and middle- income countries

**DOI:** 10.1186/1741-7015-11-71

**Published:** 2013-03-14

**Authors:** Raymond Hutubessy

**Affiliations:** 1 Initiative for Vaccine Research, World Health Organization, 20 Avenue Appia, CH-1211, Geneva, Switzerland

## Introduction

**Figure 1:**
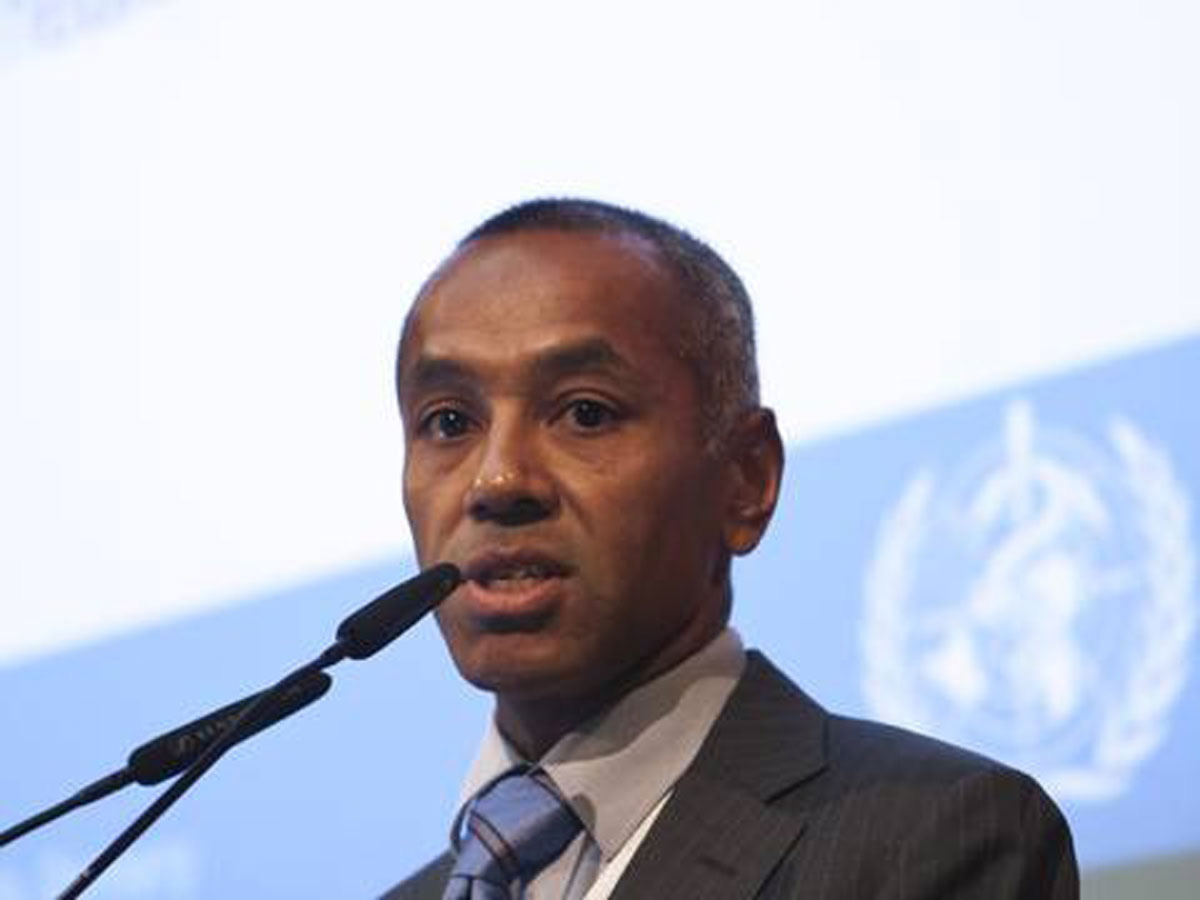
**Raymond Hutubessy**. (http://www.worldhealthsummit.org/the-summit/visuals/photo-gallery/2012/whs-monday-23rd/main-hall.html)

Raymond Hutubessy is a senior health economist affiliated to the Immunization, Vaccines and Biologicals (IVB) Department of the World Health Organization (WHO), and is the executive secretary of the WHO Immunization and Vaccines related Implementation Research Advisory Committee (IVIR-AC). His main research interests focus on economic and financial analyses of vaccine introduction decisions in low- and middle-income countries (LMICs). In this Q&A, he will discuss the importance of this work in relation to the global context of vaccine introduction decisions (Figure 1).

### 1) What is the importance of conducting economic analyses for vaccine introduction decisions?

Vaccines for prevention of communicable diseases have been shown to be extremely effective in terms of health outcomes. Therefore, conducting economic analyses to get the most value for money from vaccine introduction decisions is of high importance; evidence and information resulting from these analyses are not the only input to the decision-making process for vaccine introduction decisions, but they are important ones.

The relationship between health and economic growth is one of the cornerstones of development economics: health status is determinant of productivity that can be shown to influence economic growth. Specifically, vaccines have a broader value in terms of their indirect effects (for example, herd immunity) and other externalities (for example, improvements in the cognitive development of children, higher school attendance and attainment, macroeconomic impact). Therefore, in addition to the traditional economic appraisals for vaccine introduction decisions it is useful to policy makers and other stakeholders involved with vaccine introduction decisions to demonstrate the broader added value of vaccines and investments in health in general.

Economic appraisals address different key issues with regard to decisions on vaccine introduction. These appraisals range from priority-setting issues across vaccines and other competing health interventions, to affordability and budget impact analysis, and costing and financing issues with regard to the introduction decisions of immunization programs. For these different policy questions, different analytical tools are available, such as cost-effectiveness analyses, costing studies, budget impact and optimization analysis.

### 2) What are the main issues that should be considered?

First, because many economic evaluations are based on analytical decision tools such as mathematical infectious disease models, costing tools, decision trees models and so on, transparency is needed on the choice of the modeling methodologies, parameters and country data used and assumptions made by the analyst. Standardization of methods of cost-effectiveness is therefore needed and analysts in the field should adhere to these guides. This allows users to make comparisons of different study results by different groups. The WHO, in addition to other organizations, has developed several guidelines on economic evaluations in health, and vaccines and immunization programs in particular.

Second, to be relevant, local decision makers have country-specific policy questions and therefore need contextualized study results driven by specific country data and information with regard to demographics, epidemiological and economic data, and local needs. However, this does not mean that for each country (in my field of work, this primarily involves LMICs), economists and analysts need to start from scratch - they can build on work from other groups who often put their models, including the computer program codes, in the public domain.

In light of this, efforts should be put into the collection of local data and building local technical capacity in LMICs so that they are able to perform their own analysis and interpret their own results with the aim of increasing local ownership of the evidence generated. The WHO, along with partners i.e. Pan American Health Organization (PAHO), Agence de Médicine de Préventive (AMP), Program for Appropriate Technology in Health (PATH), Sabin Vaccine Institute and USA Centers for Disease Control and Prevention, recently started the ProVac International Working Group to promote the use of economic analysis for vaccine introduction decisions in LMICs.

### 3) Are there differences in conducting economic analyses for vaccine introduction decisions between higher income versus resource-limited settings?

In principle, the methods applied and tools used are similar in higher income versus resource-limited settings. However, because country demographics, disease burden, epidemiological and socioeconomic background, and health systems and infrastructure differ, the methods of measurement and valuation and the interpretation hence key drivers of results of economic evaluations will also differ.

For example, the price at which human papillomavirus (HPV) vaccination is considered to be cost-effective is heavily dependent on HPV prevalence and the existing local cervical cancer services and the ceiling cost-effectiveness or cost-effectiveness threshold (that is, societies' willingness to pay for an additional health gain). In high income countries, access to health care services is better and delivery systems of vaccines to reach adolescent girls are more advanced than in LMICs. As a result, the affordability question in such resource-rich scenarios focused around the relatively high public market prices of HPV vaccines, which may go up to 150 USD per dose.

By contrast, in countries eligible for the Global Alliance for Vaccines and Immunization's (GAVI Alliance) support, the manufacturers of one of the vaccines have offered an indicative price of 5 USD per dose. This is a 64% reduction on the lowest public prices. As a result, rather than the vaccine price *per se*, it is the securing of the delivery costs to get the vaccine from the port of entry to those girls in need that has become a main barrier in many of these countries. This barrier is in addition to other capacity issues, such as the existing delivery infrastructure being already over-stretched with traditional Expanded Programme on Immunization vaccines and other competing new vaccines, such as rotavirus and pneumococcal vaccines.

### 4) Are there any specific ways in which these economic analyses have improved clinical outcomes?

In my opinion, economic analyses have helped to increased the level of health that health care spending can buy, and hence have aided promotion of the use of effective and cost-effective health interventions. For example, by identifying barriers and uptake issues of vaccine introduction decisions, economic analyses have contributed to bridge the link between the evidence on theoretical vaccine efficacy and real-life effectiveness. Another example is that economic analyses do not just appraise vertical disease programs but more often also have an integrated and combined disease program perspective. This has the potential to account for synergistic effects of disease programs and therefore will improve the overall public health impact.

### 5) Where can I find more information?

WHO and other partners have vaccine specific and generic websites on health economic information and initiativies. In addition, WHO published several key documents on vaccine economics.

**WHO Initiative for Vaccine Research (IVR) **[http://www.who.int/vaccine_research/implementation/health_economics/en/index.html]

**CHOosing Interventions that are Cost Effective (WHO-CHOICE) **[http://www.who.int/choice/en/]

**WHO guide for standardization of economic evaluations of immunization programmes **[http://whqlibdoc.who.int/hq/2008/WHO_IVB_08.14_eng.pdf]

**Pan American Health Organization (PAHO ProVac) **[http://new.paho.org/provac/]

**International Health Economics Association **[https://www.healtheconomics.org/]

Jit M, Levin C, Brison M, Levin A, Resch S, Berkhof J, Kim J, Hutubessy R: **Economic analyses to support decisions about HPV vaccination in low- and middle-income countries: a consensus report and guide for analysts**. *BMC Med *2013, **11**:23.

Hutubessy R, Levin A, Wang S, Morgan W, Ally M, John T, Broutet N: **A case study using the United Republic of Tanzania: costing nationwide HPV vaccine delivery using the WHO Cervical Cancer Prevention and Control Costing Tool**. *BMC Med *2012, **10**:136.

Deogaonkar R, Hutubessy R, van der Putten I, Evers S, Jit M: **Systematic review of studies evaluating the broader economic impact of vaccination in low and middle income countries**. *BMC Public Health *2012, **12**:878.

Postma MJ, Jit M, Rozenbaum MH, Standaert B, Tu HA, Hutubessy RC: **Comparative review of three cost-effectiveness models for rotavirus vaccines in national immunization programs; a generic approach applied to various regions in the world**. *BMC Med *2011, **9**:84.

Hutubessy R, Henao AM, Namgyal P, Moorthy V, Hombach J: **Results from evaluations of models and cost-effectiveness tools to support introduction decisions for new vaccines need critical appraisal**. *BMC Med *2011, **9**:55.

Jit M, Demarteau N, Elbasha E, Ginsberg G, Kim J, Praditsitthikorn N, Sinanovic E, Hutubessy R: **Human papillomavirus vaccine introduction in low-income and middle-income countries: guidance on the use of cost-effectiveness models**. *BMC Med *2011, **9**:54.

Chaiyakunapruk N, Somkrua R, Hutubessy R, Henao AM, Hombach J, Melegaro A, Edmunds JW, Beutels P: **Cost effectiveness of pediatric pneumococcal conjugate vaccines: a comparative assessment of decision-making tools**. *BMC Med *2011, **9**:53.

## Author information

RH is a staff member of the World Health Organization. The views expressed in this article is that of the author and do not necessarily represent the views of the World Health Organization.

## Pre-publication history

The pre-publication history for this paper can be accessed here:

http://www.biomedcentral.com/1741-7015/11/71/prepub

